# Awareness, knowledge, use, and attitudes toward evidence based medicine in a developing country: survey of physicians in a canton in Bosnia and Herzegovina

**DOI:** 10.3325/cmj.2015.56.558

**Published:** 2015-12

**Authors:** Mersiha Mahmić-Kaknjo, Damira Kadić, Harun Hodžić, Selvedina Spahić-Sarajlić, Elida Hadžić, Enisa Ademović

**Affiliations:** 1 Department of Clinical Pharmacology, Zenica Cantonal Hospital, Zenica, Bosnia and Herzegovina; 2 Department of Laboratory Diagnostics, Zenica Cantonal Hospital, Zenica, Bosnia and Herzegovina; 3 Department of Urology, Zenica Cantonal Hospital, Zenica, Bosnia and Herzegovina; 4 Primary Health Care Centre Zenica, Zenica, Bosnia and Herzegovina; 5 Department of Medical Supplying, Zenica Cantonal Hospital, Zenica, Bosnia and Herzegovina; 6 Department of Epidemiology and Biostatistics, Faculty of Medicine, University of Sarajevo, Sarajevo, Bosnia and Herzegovina

## Abstract

**Aim:**

To assess awareness, knowledge, use, and attitudes toward evidence-based medicine (EBM) and The Cochrane Library (CL) among physicians from Zenica-Doboj Canton (ZDC), Bosnia and Herzegovina.

**Methods:**

In this cross-sectional study, a self-administered anonymous questionnaire was sent by post to all state owned health institutions (2 hospitals and 11 Primary Health Care Institutions) in ZDC. The main outcome measures were physicians’ awareness of the Cochrane, awareness and use of CL, access to EBM databases, and access to internet at work. 358 of 559 physicians responded (63.69%).

**Results:**

23.18% of respondents stated they had access to EBM databases, but only 3.91% named the actual EBM databases they used. The question on the highest level of evidence in EBM was correctly answered by 35.7% respondents, 34.64% heard about Cochrane and 32.68% heard about CL. They obtained information about CL mostly on the internet and from colleagues, whereas the information about EBM was obtained mainly during continuous medical education.

**Conclusion:**

Although the attitudes toward EBM are positive, there is a low awareness of EBM among physicians in ZDC. Open access to the CL should be used more. Educational interventions in popularizing EBM and Cochrane are needed to raise awareness both among students and practicing physicians, and finally among lay audience.

Evidence based medicine (EBM) is described as an integration of individual clinical expertise, the best available external clinical evidence from systematic research, and individual patients’ predicaments, rights, and preferences, in making clinical decisions about their care (1,2). However in many settings there are still barriers to its implementation (3-6).

Awareness, knowledge, use, and attitudes toward EBM have been assessed worldwide (6,7). Attitudes toward EBM were mostly positive and participants welcomed the promotion of EBM (6-11). Barriers to practicing EBM differed between developing and developed countries. For example, respondents from Iran (8) reported that a major barrier was the lack of EBM training courses, while those from the Netherlands and Belgium reported limited time, attitudes, knowledge, and skills (5,12-14).

Systematic reviews with or without meta-analysis produced by The Cochrane Library (CL) are considered as the “gold standard” in EBM (15-18). Cochrane systematic reviews (CSRs) can raise the quality of health care, especially in developing countries with scarce resources. For example, CSRs have been shown to provide invaluable evidence in creating national reimbursement lists (19).

A nation-wide study among physicians in Croatia concluded that there was low awareness about EBM and the CL (30%), and additional educational interventions were required (6). Unlike Croatia, Bosnia and Herzegovina (BH) has no organized Cochrane activity (20). Our study aimed to assess the awareness, knowledge, use, and attitudes toward EBM and the CL (as the only available EBM database in BH with unrestricted access) among physicians in Zenica-Doboj Canton (ZDC), to help in the implementation of educational activities that would improve the use of EBM and the CL.

## Methods

### Study design and settings

This is a cross-sectional survey of all physicians working in primary and secondary health care institutions in ZDC. ZDC has a population of 385 067 (21) and 13 public health care institutions: 2 secondary (Zenica Cantonal Hospital and Tešanj General Hospital) and 11 primary health care institutions (Zenica, Tešanj, Visoko, Zavidovići, Kakanj, Maglaj, Žepče, Breza, Vareš, Olovo, and Usora). At the beginning of the study, there were 559 physicians working in these institutions: 304 working in hospitals and 255 in primary health care institutions (information obtained from Chamber’s Secretary, Mrs Matić-Žilo, on October 30, 2013). According to the Law on Medical Practice (22), all physicians working with patients have to become members of Medical Chambers. Questionnaires were sent by post to each institution, with three reminders. All institutions participated in the survey. The study was conducted between October 2013 and September 2014 and was approved by the Ethics Committee of ZDC Medical Chamber.

### Survey instrument

We used a 30-item self-administered questionnaire, modified from a similar study by Novak et al (6), provided by courtesy of and approved by Livia Puljak, Split School of Medicine, Croatia. The questionnaire was modified so that the questions specifically related to Croatia were left out.

Physicians were asked about the average number of patients seen per day, physicians’ need for help in making medical decisions, internet usage and access to EBM databases, attitudes, awareness, knowledge, and use of EBM, Cochrane, and the CL, their professional status, scientific degree, and age. The questionnaire was distributed to 559 physicians together with a short letter explaining the aim of the study and stating that participation in the study was voluntary and anonymous.

### Statistical analysis

Data were analyzed by Microsoft Excel 2007 and SPSS, version 13.0 (SPSS Inc., Chicago, IL, USA). Normality of distribution of continuous variables was tested using Kolmogorov-Smirnov test. Normally distributed variables are presented as frequencies and relative frequencies. Difference in distribution frequency was tested with χ^2^ test. Continuous variables with non-normal distribution are presented using median and interquartile range (IQR). Histogram was created, and when bimodal distribution was observed, it was divided empirically (23), using 50 percentile as a cut-off point. Normality of these two distributions was then tested using Kolmogorov-Smirnov test. Distributions were presented by mode, median, and IQR. *P* values ≤0.050 were considered statistically significant. Bimodal age distribution was stratified according to variables that were indicative as a cause of bimodality. All questions with binary variables were tested in relation to the first and second modal age distributions by calculating odds ratios (OR) and 95% confidence interval (CI).

## Results

**Figure 1:**
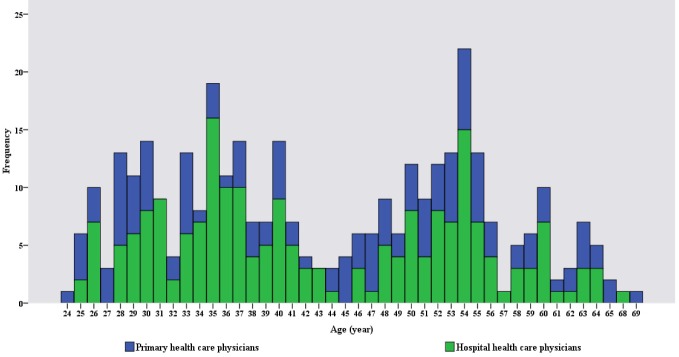
Hospital and primary health care physicians’ age.

Out of 559 questionnaires, 358 were completed and returned: 217 from hospitals and 141 from primary health care institutions. The total response rate was 63.69%, 69.97% in hospitals and 55.29% in primary health care. The sample included 55.03% (197/358) of women and 43.57% (156/358) of men (5 questionnaires had missing data) (Table 1). Median age was 42 years (IQR, 34-53 years). The data on age had a bimodal distribution (Figure 1). Median age of the first mode distribution was 34 (IQR 29-37) years, while median age of the second mode distribution was 54 (IQR 49-57) years (Table 1). Hospital physicians had a median of 20 (IQR 15-30) patients daily and primary health care physicians had 40 (IQR 30-50). The majority of physicians graduated from the Sarajevo University. Among hospital physicians, one fifth held a master’s degree and/or a PhD, which is more than among primary health physicians.

**Table 1:** Sample summary statistics and statistical test of bimodal age distribution (binned by 50 percentile as a cut- off point)

	Bimodal distribution	First mode distribution	Second mode distribution
n	343	175	168
Mean (year)	43.41	33.48	53.74
Standard deviation (year)	11.38	4.78	5.53
Range (year)	24-69	24-42	43-69
Mode (year)	35;54	35	54
Median (year)	42	34	54
Interquartile range (IQR) (year)	34-53	29-37	49-57
Test of normality	0.000	0.000	0.000

189 (52.79%) physicians assessed their knowledge of English as good or excellent and 231 (64.52%) physicians assessed their computer knowledge as good or excellent (Table 2). Almost all physicians, 354 (98.88%), stated that continuous medical education (CME) was needed.

**Table 2:** Socio-demographic characteristics of respondents

	No (%) of physicians from		
Characteristics	primary health care	hospitals	total
**Sex**			
male	60 (42.55)	96 (44.24)	156 (43.57)
female	80 (56.74)	117 (53.92)	197 (55.03)
missing data	1 (0.71)	4 (1.84)	5 (1.40)
total	141 (100.00)	217 (100.00)	358 (100.00)
**Age**			
<30	24 (17.02)	20 (9.22)	44 (12.29)
30-39	29 (20.57)	77 (35.48)	106 (29.61)
40-49	29 (20.57)	34 (15.67)	63 (17.60)
50-59	41 (29.08)	60 (27.65)	101 (28.21)
≥60	16 (11.35)	16 (7.37)	32 (8.93)
missing data	2 (1.42)	10 (4.61)	12 (3.35)
**Average No of patients seen daily**			
<20	14 (9.93)	88 (40.55)	102 (28.49)
20-39	47 (33.33)	106 (48.85)	153 (42.74)
40-59	56 (39.72)	16 (7.37)	72 (20.11)
60-79	20 (14.18)	0 (0.00)	20 (5.59)
>80	3 (2.13)	6 (2.76)	9 (2.51)
missing data	1 (0.71)	1 (0.46)	2 (0.56)
**Specialization status**			
general physician	51 (36.17)	3 (1.38)	54 (15.08)
specializing	13 (9.22)	19 (8.76)	32 (8.94)
specialist	73 (51.77)	167 (76.96)	240 (67.04)
subspecialist	2 (1.42)	28 (12.90)	30 (8.38)
missing data	2 (1.42)	0 (0.00)	2 (0.56)
**Academic degree**			
medical doctor	133 (94.33)	175 (80.65)	308 (86.03)
master of science	3 (2.13)	28 (12.90)	31 (8.66)
doctor of science	5 (3.55)	14 (6.45)	19 (5.31)
**University of graduation (medical doctor degree)**			
Sarajevo	93 (65.96)	143 (65.90)	236 (65.92)
Tuzla	31 (21.99)	34 (15.67)	65 (18.16)
other	17 (12.06)	40 (18.43)	57 (15.92)
**English language knowledge**			
no at all	6 (4.25)	6 (2.76)	12 (3.35)
superficial	22 (15.60)	22 (10.14)	44 (12.29)
basic	54 (38.30)	58 (26.73)	112 (31.28)
good	38 (26.95)	100 (46.08)	138 (38.55)
excellent	20 (14.18)	31 (14.29)	51 (14.25)
missing data	1 (0.71)	0 (0.00)	1 (0.28)
**Computer literacy**			
no at all	3 (2.13)	2 (0.92)	5 (1.40)
superficial	18 (12.77)	6 (2.76)	24 (6.70)
basic	39 (27.66)	58 (26.73)	97 (27.09)
good	62 (43.97)	113 (52.07)	175 (48.88)
excellent	19 (13.47)	37 (17.05)	56 (15.65)
missing data	0 (0.00)	1 (0.71)	1 (0.28)

Significantly more hospital physicians than primary care physicians indicated that they needed help in making medical decisions (205 [94.47%] vs 113 [80.14%], *P* < 0.001). When they needed help, hospital physicians most frequently consulted books (205 or 94.47% physicians), while primary health care physicians asked a colleague (124 or 87.94%) (Table 3).

**Table 3:** Consultations on individual patient medical issues

	No (%) of physicians from		
Question			
Help needed in reaching diagnosis or treatment option*	**primary health care**	**hospitals**	**total**
yes	113 (80.14)	205 (94.47)	318 (88.83)
no	24 (17.02)	2 (0.92)	26 (7.26)
missing data	4 (2.84)	10 (4.61)	14 (3.91)
**Source of information when help is needed in the work-up of an individual patient^†^**			
books*	101 (71.63)	205 (94.47)	306 (85.47)
colleagues	124 (87.94)	193 (88.94)	317 (88.55)
research articles*	72 (51.06)	162 (74.65)	234 (65.36)
pharmaceutical companies promotion materials	54 (38.30)	86 (39.63)	140 (39.11)
Internet*	90 (63.83)	179 (82.49)	269 (75.14)
Internet access at work*			
yes	53 (37.59)	204 (94.01)	257 (71.79)
no	85 (60.28)	7 (3.23)	92 (25.70)
missing data	3 (2.13)	6 (2.76)	9 (2.51)
Using the internet to solve medical dilemmas*			
yes	56 (39.72)	140 (64.52)	196 (54.75)
no	72 (51.06)	40 (18.43)	112 (31.28)
missing data	13 (9.22)	37 (17.05)	50 (13.97)
**Internet sources used^†^**			
Search engines (Google, etc.)*	49 (34.75)	130 (59.91)	179 (50.00)
PubMed*	20 (41.84)	77 (35.48)	97 (27.09)
Specialized EBM databases*	14 (9.93)	57 (26.27)	71 (19.83)
Other	8 (5.67)	12 (5.53)	20 (5.59)

*P < 0.001, χ^2^ test.

**^†^**more than one answer.

More hospital physicians than primary health care physicians reported having internet access at work (204 [94.01%] vs 52 [36.88%], *P* < 0.001) and using internet to solve medical dilemmas (140 [64.52%] vs 56 [39.72%], *P* < 0.001). The most frequently used information source was the internet in general (mostly searched by Google) (49 [34.75%] primary health care physicians and 130 [59.91%] hospital physicians). The second most frequently used internet source were bibliographic electronic databases, eg, PubMed (20 [41.84%] primary health care physicians and 77 [35.48%] hospital physicians).

Hospital physicians were more aware that they had access to specialized EBM databases (*P* < 0.001), although just 6 (2.76%) of them listed the CL as a specialized EBM database (Table 4). Knowledge about EBM was tested with a question about hierarchy of evidence in medicine, where approximately one third of responders answered correctly, without a significant difference between the two groups (45 [31.91%] primary health care physicians and 82 [37.79%] hospital physicians). Yet, significantly more primary health care physicians believed that case report was the highest level in the hierarchy of evidence (61 [43.26%] vs 54 (24.88%) of hospital physicians, *P* < 0.001). On the other hand, significantly more hospital physicians believed that prospective cohort studies were the highest level in the hierarchy of evidence (40 [18.43%] vs 11 [7.80%], *P* < 0.010). Significantly more primary health care physicians believed that randomized controlled trial was the highest level in the hierarchy of evidence (13 [9.22%] vs 5 [2.30%], *P* < 0.010).

**Table 4:** Responses regarding evidence based medicine (EBM)

	No (%) of physicians from		
Question			
Access to specialized EBM databases	primary health care	hospitals	total
yes†	21 (14.89)	62 (28.57)	83 (23.18)
no*	82 (58.16)	84 (38.71)	166 (46.37)
do not know	34 (24.11)	48 (22.12)	82 (22.91)
missing data†	4 (2.84)	23 (10.60)	27 (7.54)
**Which EBM databases do you use? (open ended question)^‡^**			
MEDLINE	8 (5.67)	10 (4.61)	18 (5.03)
Pubmed	6 (4.25)	12 (5.53)	18 (5.03)
Medscape	1 (0.71)	7 (3.23)	8 (2.23)
Plivamed.net	1 (0.71)	0 (0.00)	1 (0.28)
CL	6 (4.25)	6 (2.76)	12 (3.35)
Up to date	0 (0.00)	2 (0.92)	2 (0.56)
**Highest in the hierarchy of evidences**			
case report*	61 (43.26)	54 (24.88)	115 (32.12)
prospective cohort study^†^	11 (7.80)	40 (18.43)	51 (14.25)
systematic review of randomized controlled studies	45 (31.91)	82 (37.79)	127 (35.47)
single randomized controlled trial^†^	13 (9.22)	5 (2.30)	18 (5.03)
missing data	11 (7.80)	36 (16.59)	47 (13.13)
**Basic information on EBM learned during**			
undergraduate education	48 (34.04)	65 (29.95)	113 (31.56)
postgraduate education*	16 (11.35)	63 (29.03)	79 (22.07)
continuous medical education	60 (42.55)	105 (48.39)	165 (46.09)
nowhere	14 (9.93)	18 (8.29)	32 (8.94)
other^†^	3 (2.13)	19 (8.76)	22 (6.14)
specialization^†^	0 (0.00)	10 (4.61)	10 (2.79)

* *P* < 0.001, χ^2^test.

†0.001<*P* < 0.010, χ^2^test.

‡more than one answer.

The majority of participants got information on EBM through CME (Table 4). Significantly more hospital physicians as a source of information on EBM identified postgraduate education (63 [29.03%] vs 16 [11.3%], *P* < 0.001) and specialization (10 [4.6%] vs 0 [0.00%], *P* < 0.010)

Approximately a third of respondents heard about The Cochrane with no significant differences between the primary health care and hospital physicians (49 [34.75%] vs 75 [34.5%]) and there was a similar situation with the CL (46 [32.62%] vs 71 [32.72%]). The majority of both groups heard about it on the internet (31 [21.99%]) primary health care physicians vs 33 [15.21%] hospital physicians). 69 (19.27%) physicians used CL; 24 (17.02%) primary health care physicians and 45 (20.73%) hospital physicians. Only a few of them read full articles, 5 (3.55%) primary health care physicians and 13 (5.99%) hospital physicians. The most frequent point of access to the CL was home – 42 (11.73%) respondents, and 24 (6.70%) respondents used it several times a month (Table 5). 41 respondents (11.45%) believed that the CL helped sufficiently. 67 (18.72%) physicians were willing to learn more about the methodology of performing CSRs.

**Table 5:** Knowledge and usage of *The Cochrane Library (CL)*

Question	No (%) of physicians from		
Heard of The Cochrane	primary health care	hospitals	Total
yes	49 (34.75)	75 (34.5)	124 (34.64)
no	82 (58.16)	112 (51.61)	194 (54.19)
missing data	10 (7.09)	30 (13.82)	40 (11.17)
**Heard of CL**			
yes	46 (32.62)	71 (32.72)	117 (32.68)
no	43 (30.50)	61 (28.11)	104 (29.05)
missing data	52 (63.88)	85 (39.17)	137 (38.27)
**Got information on CL from:**			
books*	2 (1.42)	15 (6.91)	17 (4.75)
colleagues	17 (12.06)	27 (12.44)	44 (12.29)
research articles	8 (5.67)	25 (11.52)	33 (9.22)
leaflets of pharmaceutical companies	1 (0.71)	0 (0.00)	1 (0.28)
internet	31 (21.99)	33 (15.21)	64 (17.88)
other	7 (4.96)	15 (6.91)	22 (6.14)
missing data	75 (53.19)	102 (47.00)	177 (49.44)
**Reading in CL**			
summaries	19 (13.47)	22 (10.14)	41 (11.45)
full texts	5 (3.55)	13 (5.99)	18 (5.03)
**CL use frequency**			
fewer that once a month	5 (3.55)	11 (5.07)	16 (4.47)
once a month	3 (2.13)	3 (1.38)	6 (1.66)
several times a month	11 (7.80)	13 (5.99)	24 (6.70)
once a week	2 (1.42)	4 (1.84)	6 (1.68)
several times a week	3 (2.13)	4 (1.84)	7 (1.96)
**CL helpful in solving problems**			
not at all*	0 (0.00)	6 (2.76)	6 (1.68)
very little	5 (3.55)	3 (1.38)	8 (2.23)
helped enough	14 (6.45)	27 (12.44)	41 (11.45)
very much*	5 (3.55)	0 (0.00)	5 (1.40)
completely	0 (0.00)	1 (0.46)	1 (0.28)
**CL could help to solve problems**			
yes	15 (10.64)	18 (8.29)	43 (12.01)
no	6 (4.25)	21 (9.68)	27 (7.54)
**Interested in Cochrane systematic reviews production methodology**			
yes	24 (17.02)	43 (19.82)	67 (18.71)
no	8 (5.67)	13 (5.99)	21 (5.87)

*0.001<*P* < 0.010, χ^2^ test.

Physicians that held master’s and/or PhD degree showed significantly higher level of knowledge on the hierarchy of evidence; half of the physicians with a degree in science – 26 (52.00%) answered the question on the hierarchy of evidence correctly compared to a third of physicians without a degree in science – 101 (32.79%) (0.001<*P* < 0.010).

Since bimodal distribution of age was observed, the respondents were divided into two groups according to age: 24-42 year group and 43-69 year group. Three significant differences were observed: more physicians aged 24-42 years needed help while working (OR 2.1, 95% CI 1.07-4.13), they more frequently used the web (OR 1.63, 95% CI 1.01-2.63), and consulted web sources for help (OR 2.44, 95% CI 1.07-5.53) than those aged 43-69 years.

## Discussion

Our study showed that the awareness, knowledge, and use of EBM and CL in ZDC was low despite the fact that the most participants had positive attitude toward EBM and many of them were interested to learn about the methodology of making CSRs. The awareness of EBM in our setting (23%) was lower than that in Croatia (6) (54%), while the awareness of the CL was similar to that in Croatia, where one third of participants heard of Cochrane (6), and about half of the participants were unaware of the CL (11).

The research methods used in this study have some limitations. The response rate was similar to that in the study by Novak et al (6), but our sample was not representative of the population of physicians from the entire country, since it included physicians only from one canton. Another limitation was the use of a self-administered questionnaire, which yielded a lot of missing data, compared to the study by Novak et al, which used a telephone survey.

The bimodal age distribution found in our study, unusual for other settings, indicates that there are very few middle generation physicians. This finding can be explained by emigration of physicians during the war in BH (1992-1996), and a smaller number of physicians graduating during the war years. This means that future educational activities have to be targeted at two different groups of physicians: those who graduated before the war and those who graduated after the war.

Major barriers to practicing EBM were insufficient knowledge on EBM and insufficient internet availability. In our setting, internet availability at work was 71.79%, better than in Jordan (11) – 53.70%, but worse than in Croatia (6) – 99.1% in hospital setting and 83.4% in primary health care setting.

To be able to practice EBM, physicians need to get acquainted with EBM, Cochrane, and the CL and its use in daily practice, which is why a mandatory, vertically integrated course in research methodology should be introduced into the medical curriculum (24).

Fortunately, Wiley provides free access for middle and low income countries, which includes BH. Also, thanks to the Croatian Cochrane Branch, BH physicians have a free access to summaries translated into Croatian (*cochrane.org/hr/evidence, croatia.cochrane.org/hr*), which is one of the official languages in BH and is understood by the speakers of the Bosnian and Serbian language.

We are glad that even this study was an act of promotion of the CL, since we were repeatedly asked by the physicians about the details of the CL usage. Some of the respondents stated that this questionnaire was the first time they heard about the CL. Additionally, following the footsteps of the neighboring Croatia, we started publishing articles on Cochrane in the official journal of BH Academy of Sciences (20).

Knowledge and practice of EBM among physicians in ZDC were not very high, but the attitudes toward EBM were relatively positive. There is a need for specifically designed educational interventions that would encourage physicians to use open access to the CL, as well as experiences and materials from Cochrane Croatia. Improving internet accessibility at work is needed, especially in primary health care setting. Special attention should be paid to pregraduate and postgraduate students of medicine and physicians younger than 42, who tend to use web based resources more frequently. Integrating EBM longitudinally and vertically throughout the academic curriculum would be beneficial for promotion and application of clinical knowledge in daily practice in order to improve community health.

## References

[1] Seshia SS, Young GB (2013). The evidence-based medicine paradigm: where are we 20 years later? Part 1.. Can J Neurol Sci.

[2] Sackett DL, Rosenberg WM, Gray JA, Haynes RB, Richardson WS (1996). Evidence based medicine: what it is and what it isn't.. BMJ.

[3] Albarrak AI, Ali Abbdulrahim SA, Mohammed R (2014). Evaluating factors affecting the implementation of evidence based medicine in primary healthcare centers in Dubai.. Saudi Pharm J.

[4] Hannes K, Leys M, Vermeire E, Aertgeerts B, Buntinx F, Depoorter AM (2005). Implementing evidence-based medicine in general practice: a focus group based study.. BMC Fam Pract.

[5] Zwolsman SE, van Dijk N, Te Pas E, Wieringa-de Waard M (2013). Barriers to the use of evidence-based medicine: knowledge and skills, attitude, and external factors.. Perspect Med Educ..

[6] Novak K, Miric D, Jurin A, Vukojevic K, Aljinovic J, Caric A (2010). Awareness and use of evidence-based medicine databases and Cochrane Library among physicians in Croatia.. Croat Med J.

[7] Abeysena C, Jayawardana P, Wickremasinghe R, Wickramasinghe U (2010). Evidence-based medicine knowledge, attitudes, and practices among doctors in Sri Lanka.. J Evid Based Med..

[8] Mozafarpour S, Sadeghizadeh A, Kabiri P, Taheri H, Attaei M, Khalighinezhad N (2011). Evidence-based medical practice in developing countries: the case study of Iran.. J Eval Clin Pract.

[9] Mittal R, Perakath B (2010). Evidence-based surgery: knowledge, attitudes, and perceived barriers among surgical trainees.. J Surg Educ.

[10] Barghouti F, Halaseh L, Said T, Mousa AH, Dabdoub A (2009). Evidence-based medicine among Jordanian family physicians: awareness, attitude, and knowledge.. Can Fam Physician.

[11] Al Omari M, Khader Y, Jadallah K, Dauod AS, Al-Shdifat AA, Khasawneh NM (2009). Evidence-based medicine among hospital doctors in Jordan: awareness, attitude and practice.. J Eval Clin Pract.

[12] Zwolsman S, te Pas E, Hooft L, Wieringa-de Waard M, van Dijk N (2012). Barriers to GPs' use of evidence-based medicine: a systematic review.. Br J Gen Pract.

[13] van Dijk N, Hooft L, Wieringa-de Waard M (2010). What are the barriers to residents' practicing evidence-based medicine? A systematic review.. Acad Med.

[14] Heselmans A, Donceel P, Aertgeerts B, Van de Velde S, Ramaekers D (2009). The attitude of Belgian social insurance physicians towards evidence-based practice and clinical practice guidelines.. BMC Fam Pract.

[15] The Cochrane Collaboration. About us. 2014. Available from: http://www.cochrane.org/about-us. Accessed: December 26, 2015.

[16] Moja LP, Telaro E, D'Amico R, Moschetti I, Coe L, Liberati A (2005). Assessment of methodological quality of primary studies by systematic reviews: results of the metaquality cross sectional study.. BMJ.

[17] The Cochrane Collaboration. The Cochrane Libraray. Available from: http://www.thecochranelibrary.com/view/0/AboutTheCochraneLibrary.html. Accessed: December 26, 2015.

[18] Borlawsky T, Friedman C, Lussier YA (2006). Generating executable knowledge for evidence-based medicine using natural language and semantic processing.. AMIA Annu Symp Proc.

[19] Mahmic-Kaknjo M, Marusic A (2015). Analysis of evidence supporting the Federation of Bosnia and Herzegovina reimbursement medicines lists: role of the WHO Essential Medicines List, Cochrane systematic reviews and technology assessment reports.. Eur J Clin Pharmacol.

[20] Mahmic-Kaknjo M, Puljak L, Markotic F, Fidahic M, Muhamedagic L, Zakarija-Grkovic I (2015). Cochrane and its prospects in Bosnia and Herzegovina: Relying on Cochrane Croatia. Acta Medica Academica.

[21] Agency for Statistics of Bosnia and Herzegovina. Preliminary results of the 2013 Census of Population, Households and Dwellings in Bosnia and Herzegovina, first release. 2013 [cited 2014 May 21st]; Available from: http://www.bhas.ba/obavjestenja/Preliminarni_rezultati_bos.pdf. Accessed: December 26, 2015.

[22] Law on Medical Practice. Official Gazette of Federation of Bosnia and Herzegovina, No 56, July 19th. 2013.

[23] Frankland BWZ (2002). B.D. Quantifying Bimodality Part I: An easily implemented method using SPSS.. Journal Of Modern Applied Statistical Methods..

[24] Marusic A, Malicki M, Sambunjak D, Jeroncic A, Marusic M (2014). Teaching science throughout the six-year medical curriculum: two-year experience from the University of Split School of Medicine, Split, Croatia. Acta Medica Academica.

